# Validity and reliability of a novel 3D scanner for assessment of the shape and volume of amputees’ residual limb models

**DOI:** 10.1371/journal.pone.0184498

**Published:** 2017-09-08

**Authors:** Elena Seminati, David Canepa Talamas, Matthew Young, Martin Twiste, Vimal Dhokia, James L. J. Bilzon

**Affiliations:** 1 Department for Health, University of Bath, Bath, United Kingdom; 2 CAMERA Centre, University of Bath, Bath, United Kingdom; 3 Department of Mechanical Engineering, University of Bath, Bath, United Kingdom; 4 School of Health Sciences, University of Salford, Salford, United Kingdom; 5 United National Institute for Prosthetics & Orthotics Development (UNIPOD), University of Salford, Salford, United Kingdom; Northwestern University, UNITED STATES

## Abstract

**Background:**

Objective assessment methods to monitor residual limb volume following lower-limb amputation are required to enhance practitioner-led prosthetic fitting. Computer aided systems, including 3D scanners, present numerous advantages and the recent Artec Eva scanner, based on laser free technology, could potentially be an effective solution for monitoring residual limb volumes.

**Purpose:**

The aim of this study was to assess the validity and reliability of the Artec Eva scanner (practical measurement) against a high precision laser 3D scanner (criterion measurement) for the determination of residual limb model shape and volume.

**Methods:**

Three observers completed three repeat assessments of ten residual limb models, using both the scanners. Validity of the Artec Eva scanner was assessed (mean percentage error <2%) and Bland-Altman statistics were adopted to assess the agreement between the two scanners. Intra and inter-rater reliability (repeatability coefficient <5%) of the Artec Eva scanner was calculated for measuring indices of residual limb model volume and shape (i.e. residual limb cross sectional areas and perimeters).

**Results:**

Residual limb model volumes ranged from 885 to 4399 ml. Mean percentage error of the Artec Eva scanner (validity) was 1.4% of the criterion volumes. Correlation coefficients between the Artec Eva and the Romer determined variables were higher than 0.9. Volume intra-rater and inter-rater reliability coefficients were 0.5% and 0.7%, respectively. Shape percentage maximal error was 2% at the distal end of the residual limb, with intra-rater reliability coefficients presenting the lowest errors (0.2%), both for cross sectional areas and perimeters of the residual limb models.

**Conclusion:**

The Artec Eva scanner is a valid and reliable method for assessing residual limb model shapes and volumes. While the method needs to be tested on human residual limbs and the results compared with the current system used in clinical practice, it has the potential to quantify shape and volume fluctuations with greater resolution.

## Introduction

The post-operative phase following limb amputation is characterised by rapid residual limb volume reduction due to decreased post-surgical oedema and muscle atrophy. The range of volume reduction, 6-months post-surgery, varies between 17% and 40% of the original volume and appears to be characterised by a negative exponential function of time [1–3]. The transition from acute clinical rehabilitation to stable long-term recovery occurs 12–18 months post-operatively [4] and currently there is no definitive method for establishing when the residual limb volume has stabilised [2, 3]. In addition, mature residual limbs (i.e. >18-months post amputation) are still subject to short term volume changes (diurnal changes range between -3.5% and +10.9% [5–7]). Prosthetists engage in labour-intensive processes to modify sockets using manual methods, and fitting problems can eventually result in expansive and time consuming socket-prosthesis adjustments/replacements and low quality of life [8]. Management and assessment of residual limb volume is important because it affects decisions regarding: i) timing of fit of the first prosthesis (i.e. when to switch from a temporary to a definitive prosthetic socket); ii) design of a prosthetic socket/liner that can best transmit loading forces, minimising discomfort through offering an ‘ideal fit’ without compromising easy donning/doffing of the prosthesis (if high stresses develop where the skin is of low load-tolerance, then the normal, shear, and frictional forces can compound and lead to pain, surface abrasions, deep skin breakdown or even deep tissue injury) and; iii) prescription of accommodation strategies for daily volume fluctuations. Furthermore, by building a database of residual limb volume changes, which include details of patient background and lifestyle behaviours, predictive modelling work can be undertaken to help streamline the process of prosthetic refitting for individuals undergoing routine volume change.

Many techniques for the measurement of residual limb volume have been described, using patient residual limbs and models. Not all methods are suitable for clinical use, either because they lack necessary resolution for volume measurement and/or they are unable to detect changes in residual limb shape [4]. Anthropometric measures [9, 10] are practical and acceptable for macroscopic residual limb changes, but they have a poor inter- and intra-observer repeatability and lack the precision necessary to establish the threshold for stabilisation. Water immersion methods [11] have lower variability, but cannot be used when open wounds are present or on patients with bilateral leg amputations. MRI [12], ultrasound measurement [13, 14] and computed tomography (CT) scans [15, 16] have been described in the literature, and they have the potential to detect volume, shape and internal structures of the residual limb. However, they are not used in clinical practice, since they are very expensive, they can introduce some distortions due to the position of the patients (MRI and CT scans) and some of them (ultrasounds and MRI) are very time consuming (between 10 and 13 minutes of scanning time). Laser scanning methods, including CAD/CAM systems [1, 9, 17–21], have been introduced to aid the manufacture of prosthetic sockets, reducing fitting errors, fabrication time and overall costs. Some of these techniques have shown promise, with the Omega Tracer scanner being the method of choice for current clinical practice in the UK [2, 9]. Repeatability coefficients for this system appear relatively high and range from 45 ml (~5% of volume) when scanning residual limb models [9] to 129 ml (~13% of volume) when scanning human transtibial residual limbs [22]. A recent study evaluated the accuracy of the imaging/acquisition process of three new surface 3D scanners and suggested that the VIUScan marker assisted laser scanner was the most accurate for determining residual limb model volume and shape. The systematic bias/error reported between the VIUScan scanner and the criterion was lower than 1%, but they used a single 3D printed transtibial model of known geometry as a gold standard, without considering the effect of different model sizes [23]. The method revealed estimated repeatability coefficients <45 ml for within and between observer assessments. However, a full comparison is limited because of the statistical methods adopted. Furthermore, as with many other similar studies, this method has not yet been tested on human residual limbs.

New CAD/CAM technologies are emerging, with new features that can help prosthetists and practitioners in decision-making regarding the timing and design of prosthetic sockets. The Artec Eva scanner (Artec Group, Luxembourg, Luxembourg) is a state-of-the art technology for 3D surface scanning. It uses laser free structured light scanning technology and has already been successful in human applications [24–26], but it has not been used for the assessment of amputee residual limb shape or volume. It is quick to use, can accommodate some target movement and is able to capture geometry and texture/colour information, facilitating anatomical features detection, which eliminates the need for reference targets or markers to be placed on the limb. Before adopting this new system in clinical practice, it is necessary to determine its accuracy in terms of validity and reliability with residual limb models. As residual limb models are not prone to movement artefact, the technical measuring error of the equipment and of the observer can be independently assessed.

The aim of this study was therefore to assess the accuracy (validity and reliability) of the Artec Eva scanner for estimating the volume, shape and size of transtibial and transfemoral residual limb models. In order to be clinically meaningful, the Artec Eva scanner would need to demonstrate a mean percentage error (validity) of <2% compared to the Romer scanner (criterion measure). In order to be considered reliable, it would need to elicit intra and inter-rater repeatability coefficients of <5%. Validity threshold has been determined, after considering the most accurate methods presented in the literature [4, 23]. The repeatability threshold has been set, in part, based on the methods currently used in clinical practice [9] and, also, the desire to detect smaller but meaningful acute residual limb changes (short term changes, diurnal changes or changes due to postdoffing).

## Materials and methods

In this study, ten residual limb models were scanned by three independent observers, each on three separate occasions, using two different scanners (i.e. 180 scans), over a 4 months’ period (May—August 2016). The models were selected from anonymous transtibial (n = 5) and transfemoral (n = 5) amputees (Table 1) to evaluate a large range of representative shapes and volumes. The models were distributed via prosthetics centres. They were manufactured using a standard carver for milling out foam models and a standard negative plaster-bandage wrap cast as a mould in which liquid plaster could set for plaster models.

**Table 1:** Residual limb models characteristics.

Model	Level	Material	Romer scanner (ml)	Artec scanner (ml)
1	TF	foam	4277 (3)	4316 (7)
2	TF	foam	2332 (4)	2362 (10)
3	TT	foam	1782 (1)	1807 (7)
4	TF	plaster	4019 (3)	4053 (12)
5	TF	plaster	3003 (2)	3030 (5)
6	TF	foam	2930 (3)	2969 (8)
7	TT	foam	2606 (4)	2643 (5)
8	TT	foam	1326 (1)	1352 (3)
9	TT	foam	1529 (2)	1555 (2)
10	TT	foam	869 (1)	887 (4)

Level of amputation (transfemoral—TF or transtibial—TT), type of material (foam or plaster) and mean volumes calculated with the Romer and the Artec scanners. The values reported in column 4 and 5 represent the mean volume of 9 trials (three operators and three trials for each scanner). Standard deviation is indicated in brackets.

### Measurements systems

**Fig 1:**
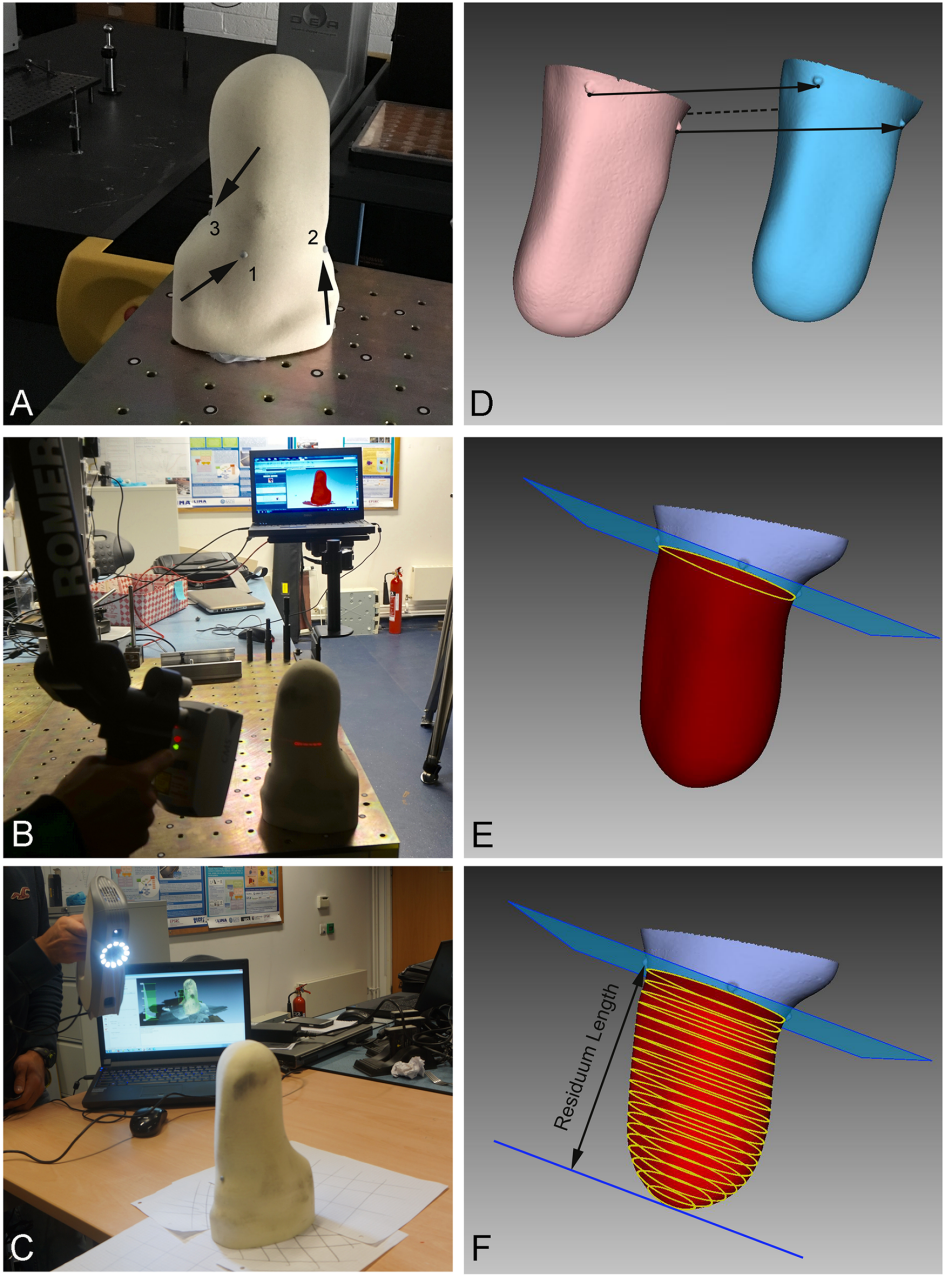
Residual limb scanning and processing procedures. A) Example of transtibial residual limb with the three anatomical markers; B) Romer scanning conditions; C) Artec Eva scanning conditions; D) Romer (pink) and Artec Eva (blue) 3D models prior to alignment; E) aligned models with the plane 0, defined as the plane passing through the distal border of the three reference points; F) CSAs along the residual limb.

The use of high precision and resolution laser scanner has been suggested for evaluation of new scanning systems [4]. For this reason, we used the Romer high precision and resolution scanner (Romer scanner, CMS108, Hexagon, UK) as the criterion measure to validate the ‘trueness’ of the Artec Eva scanner (practical measure). The Romer scanner is a powerful tool integrated with a Romer coordinate measuring arm that comprises different rotation axes to allow freedom of movement. It uses a laser line to reconstruct the 3D model (Fig 1B) with an accuracy of about 0.04 mm [27]. In contrast, the Artec Eva (practical measure) is relatively small and uses regular flash bulb technology, illuminating the object with patterns of stripes by normal visible light to reconstruct 3D data from the surface with a reported accuracy of 0.5 mm [25].

### Experimental design and data collection

Three independent observers were trained to use both the Artec Eva and Romer scanners. Prior to data collection, they completed two 2-hour familiarisation sessions, using both scanners. Ten different sessions were organised to measure each of the ten selected residual limb models. During each session, each model was measured three times by each observer with the two different scanners. This resulted in a total of 18 measurements (2 scanners × 3 observers × 3 repetitions) per model/session. The observers performed the measurements in randomised order (both observer sequence and scan sequence), with a 10-min break between each scan. Time per scan was between 1 and 3 minutes for the Artec Eva and Romer scanners, respectively.

Prior to scanning, three 4 mm diameter hemispherical adhesive markers made of soft rubber were placed on the 3D surface of each residual limb model to approximately identify three anatomical landmarks for transfemoral (greater trochanter, Scarpa’s triangle and ischial tuberosity) and transtibial (tibial crest, fibula head and popliteal fossa) models (Fig 1A). The distal borders of these markers were used to determine a plane used as the proximal end of each scan. Each model was placed on a metrology table for the Romer scans and on a normal table for the Artec Eva scans, with the distal end of the residual limb pointing upward (Fig 1B and 1C).

### Data processing

Artec Eva and Romer data files were processed using the same software used for data collection: Artec Studio 9.2 (Artec Group, Luxembourg, Luxembourg) and Geomagic Studio 2014 (Geomagic—3D Systems, USA). Both the Artec Eva and Romer mesh models were exported and aligned manually in the same reference system x, y and z, using a graphical user interface according to the positions of the anatomical markers on the model (Fig 1D). The volume of each residual limb model was calculated using the distal end to the proximal end of the residual limb as indicated by the plane and defined by the three anatomical markers (Fig 1E). Parallel to this first plane 19 other planes were defined across the residual limb volume, obtaining a set of 20 parallel different sections at intervals of 5% across the residual limb length, with the first section (i.e. 0%) indicating the first proximal section of the residual limb model. For each section created by the 20 planes the relative Cross Sectional Area (CSA) and the perimeter (PE) were calculated (Fig 1F).

To assess residual limb model geometrical differences between the two scanners, the Root Mean Square Error (RMSE) between each pair of aligned scans was calculated. In addition, assuming the residual limb to be confined in a bounding box, residual limb sizes (width, depth and length) along three axes (x, y, and z, respectively) were calculated. Body Centre of Mass (BCOM) coordinates were calculated assuming the material of the models to be homogenous. For these calculations, each volume was processed using the Compute geometric measures filter [28] in Meshlab software.

### Statistical analysis

Accuracy of the Artec Eva scanner was assessed in terms of validity (trueness) and reliability (precision) using the statistical approach suggested by Hopkins, which was used to assess the validity and reliability of DEXA imaging methods [29, 30]. The following scanning variables were considered: residual limb volume, residual limb sizes (width, depth and length) along the three different axes (x, y, and z, respectively), BCOM coordinates, CSA and PE for each of the 20 levels of the residual limb length, where level 0 was defined as the plane passing through the distal border of the three anatomical/reference points. To ensure normality of the sampling distribution, each measurement was log transformed before analysis and back transformed after analysis [31, 32]. Log transformation was necessary to ensure uniformity of error, particularly where larger values of the original variable have greater absolute but a similar relative (%) error. Log transformation was not applied to the BCOM variables, since they were expressed in the relative Romer reference system.

#### Validity

Validity of the Artec Eva scan was assessed using a previously published method [33], where the Romer scanner was considered as the criterion and the Artec Eva scanner as the practical measurement system. Limits of agreement were calculated using methods described by Bland-Altman [30]. The overall bias and 95% limits of agreement were calculated and Pearson correlation coefficient and Coefficient of Variation (CV) were determined. Modified Bland and Altman plots were used to show the Artec Eva validity as percentage change, when considering either only transtibial or only transfemoral residual limb models. We considered using a mixed-model analysis of variance method to ensure that assumptions of sample independence were not violated, but the differences in results were negligible.

#### Reliability

To quantify the intra-rater variability (repeatability) of the Artec Eva scanner, data from repeated scans were assembled in three groups (one for each scan repetition). The same data were assembled in three different groups (one for each observer) to quantify the inter-rater variability (reproducibility). Mean and standard deviation values were calculated for each sub-group. Data from repeated scans were used to calculate change in the mean (the mean difference between the repeated scan results), typical error of the measurements (TEMs; standard deviation of the difference scores of all the scans in the group divided by √2), and Intra-class Correlation Coefficients (ICCs) for all scan parameters, using a published method [34], with a 95 percent Confidence Interval (CI). The type of ICC produced by Hopkins’ analysis of validity and reliability [34] for intra- and inter-rater reliability, was ICC(3,1) [33]. According to Bland and Altman [31] the within—subject standard deviation (or standard error of the measurement [31]), represented by the TEMs, was used to calculate the intra-rater variability (repeatability coefficients) and inter-rater volume variability (reproducibility coefficient) as 1.96√2TEMs as reported in previous studies regarding residual limb volume repeatability [9, 22, 31].

#### Reporting

This article was prepared in accordance with the checklist for Strengthening the Reporting of Observational Studies in Epidemiology (STROBE), which is provided as a supplementary file (S1 Table).

## Results

The three observers collected 60 scans each, during ten different sessions, and the measured volumes ranged from 885 ml to 4399 ml (Table 1).

### Validity

**Fig 2:**
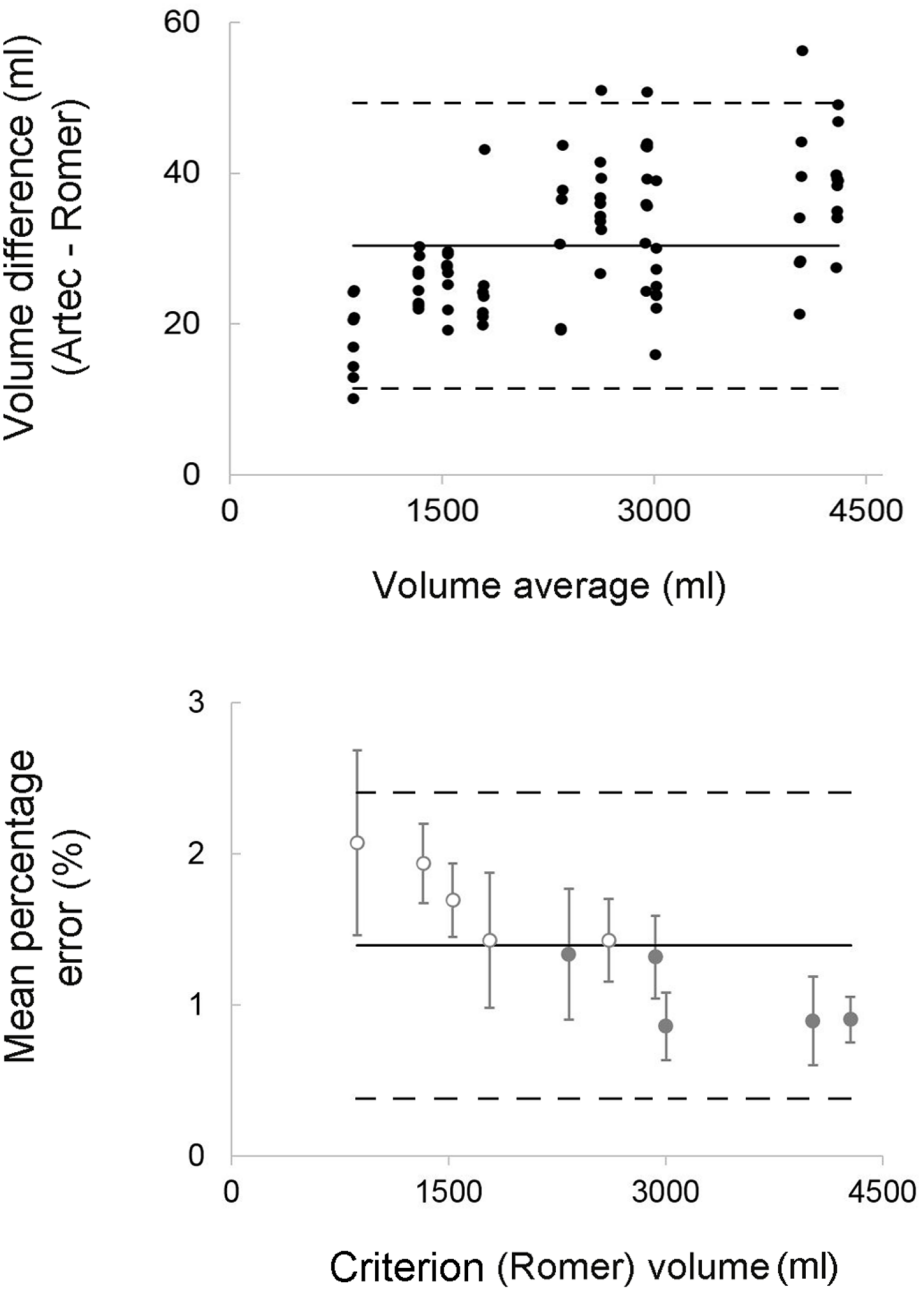
Scanners’ agreements. Top panel: Bland-Altman plots for model volumes calculated with the criterion (Romer scanner) and the practical (Artec Eva scanner) measurement system; Bottom panel: modified Bland-Altman plots displaying the error of volumes measured with the practical (Artec Eva) scanner expressed as a percentage of the Romer scanner volumes (average between trials). The open circles represent transtibial models, while the full circles represent transfemoral models. The dashed lines indicate the upper and lower 95% limits of agreements.

**Fig 3:**
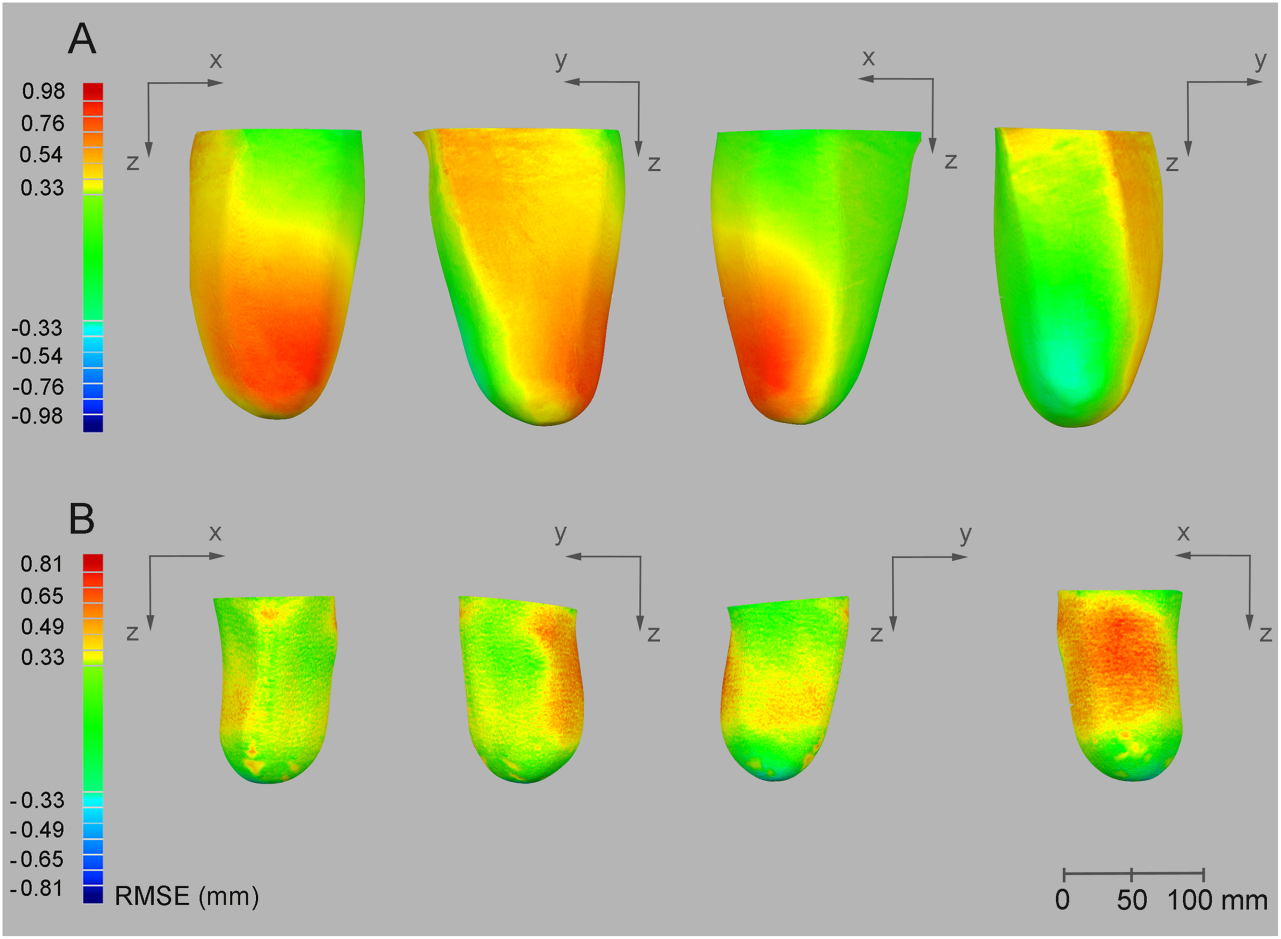
RMSE results. Example comparison of the same model collected with the 2 different scanners from the same observer for a right-sided trans-femoral models (A) and a left-sided transtibial model (B). RMSE differences are indicated by the coloured scale on the left (red positive mean values indicate that the Artec Eva scanner measured a bigger volume). From left to right: anterior view, lateral view, medial view and posterior view of the residual limb model.

Volume measurements showed a consistent tendency for the volumes obtained with the Artec Eva scanner to exceed (~30 ml) values obtained with the Romer scanner. This bias corresponded to 1.4% of the actual volumes with most differences between measurements by two methods falling within 1% of limits of agreements (Fig 2). Similarly, model size measurements (i.e. width, depth and length), confirmed the overestimation of the Artec Eva scanner relative to the Romer, even though the bias was always lower than 1 mm and with 1% of Limits of Agreement. BCOM coordinates estimation showed low bias values (< 1mm) with the highest variability observed for the vertical coordinate (z) (Table 2). The average RMSE values calculated in three dimensions between Artec Eva and Romer scans ranged from 0.23 to 0.65 mm, with the Artec Eva scanner presenting higher values than the Romer scanner (Fig 3).

**Table 2:** Validity results.

	Overall Bias		Limits of Agreements			
	Absolute	Relative (%)	Absolute	Relative (%)	Pearson CC	CV (%)
**Volume**	30.4 (28.3; 32.5) ml	1.40 (1.28; 1.51)	18.9 ml	1.01	0.99	0.34 (0.29; 0.40)
**Residuum Size**						
**Width (x)**	0.49 (0.39; 0.59) mm	0.36 (0.29; 0.43)	0.91 mm	1.01	0.99	0.32 (0.28; 0.38)
**Depth (y)**	0.47 (0.38; 0.55) mm	0.35 (0.29; 0.41)	0.77 mm	1.01	0.99	0.24 (0.21; 0.28)
**Length (z)**	0.38 (0.26; 0.50) mm	0.14 (0.10; 0.19)	1.09 mm	1.00	0.99	0.20 (0.17; 0.23)
**BCOM**						
**x**	-0.01 (-0.04; 0.03) mm	-	0.32 mm	-	0.99	-
**y**	0.02 (-0.04; 0.07) mm	-	0.48 mm	-	0.99	-
**z**	0.23 (0.05; 0.41) mm	-	1.63 mm	-	0.99	-

Validity results are presented in terms of overall bias (absolute and relative values), limits of agreements, Pearson Correlation Coefficient (CC) and Coefficient of Variation (CV). Values in brackets represent the 95% Confidence Limits.

**Fig 4:**
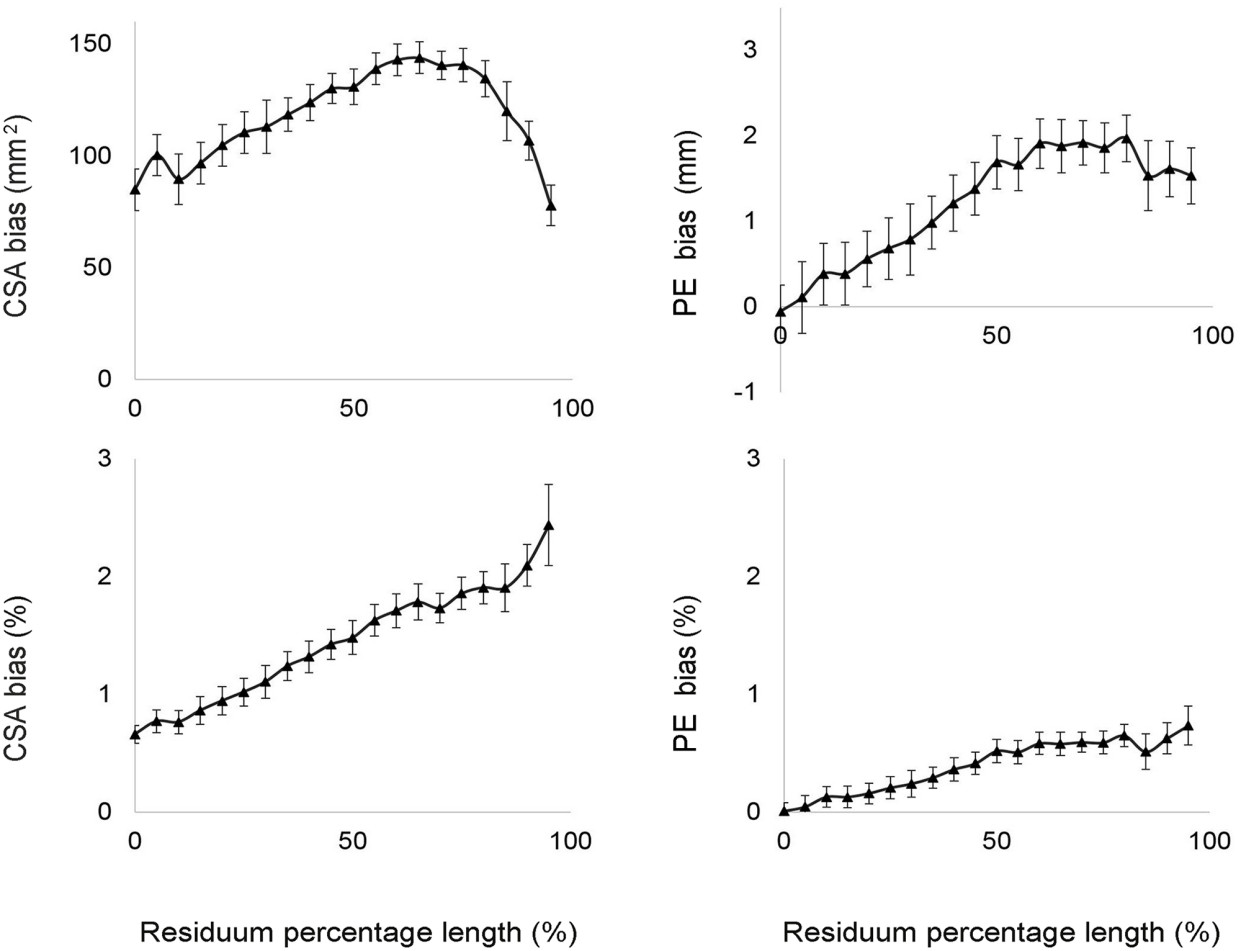
Residual limb shape validity results. The graphs represent the bias and limits of agreement for CSAs and PE calculated for each section along the residual limb model, and expressed both in absolute units and as a relative percentage of the Romer scanner’s original measure (0% indicates the first proximal section of the residual limb model).

The bias of the CSAs and of the PE increased along the longitudinal length of the residual limb models, reaching differences close to 2% for the CSA and to 1% for the PE at the distal end of the model (Fig 4).

### Reliability

The Artec Eva scanner showed high levels of intra- and inter-rater reliability, with ICCs greater than 0.90 as it was for the criterion measurement. Intra- and inter-rater repeatability coefficients for volume measurements were 13.9 and 18.6 ml for the Artec Eva scanner (respectively 0.5% and 0.7% of the average volumes), with TEMs slightly higher for the inter-rater variability (Table 3). Good reliability was also evident for model size and BCOM estimation with errors always lower than 0.2% for the model size and lower than 1 mm for the BCOM.

**Table 3:** Reliability results.

**Intra-rater**					
		**Typical Error of Measurement**			
	**Change in Mean**	**Absolute**	**Relative (%)**	**Reliability**	**ICC**
**Volume**	0.82 (-1.86; 3.51) ml	5.03 (4.20; 6.44) ml	0.20 (0.17; 0.26)	13.94 ml	0.99
**Residuum Size**					
**Width (x)**	0.01 (-0.07; 0.10) mm	0.16 (0.12; 0.22) mm	0.11 (0.09; 0.15)	0.43 mm	0.99
**Depth (y)**	-0.06 (-0.18; 0.06) mm	0.25 (0.29; 0.35) mm	0.14 (0.11; 0.20)	0.69 mm	0.99
**Length (z)**	0.05 (-0.06; 0.16) mm	0.21 (0.16; 0.29) mm	0.08 (0.06; 0.12)	0.58 mm	0.99
**BCOM**					
**x**	-0.02 (-0.07; 0.03) mm	0.11 (0.08; 0.15) mm	-	0.29 mm	0.99
**y**	0.05 (-0.04; 0.14) mm	0.19 (0.15; 0.27) mm	-	0.54 mm	0.99
**z**	0.06 (-0.06; 0.18) mm	0.30 (0.24; 0.42) mm	-	0.83 mm	0.99
**Inter-rater**					
**Volume**	-0.54 (-4.14; 3.07) ml	6.69 (5.54; 8.50) ml	0.30 (0.25; 0.38) %	18.55 ml	0.99
**Residuum Size**					
**Width (x)**	0.03 (-0.11; 0.17) mm	0.27 (0.22; 0.35) mm	0.19 (0.16; 0.25) %	0.75 mm	0.99
**Depth (y)**	0.02 (-0.13; 0.16) mm	0.26 (0.22; 0.34) mm	0.16 (0.13; 0.20) %	0.73 mm	0.99
**Length (z)**	0.02 (-0.10; 0.14) mm	0.22 (0.18; 0.29) mm	0.09 (0.07; 011) %	0.62 mm	0.99
**BCOM**					
**x**	0.00 (-0.02; 0.01) mm	0.03 (0.03; 0.04) mm	-	0.09 mm	0.99
**y**	0.01 (-0.01; 0.04) mm	0.05 (0.04; 0.06) mm	-	0.12 mm	0.99
**z**	-0.05 (-0.17; 0.08) mm	0.29 (0.24; 0.37) mm	-	0.79 mm	0.99

Intra-rater reliability (upper panel) and inter-rater reliability (lower panel) results are presented in terms of Change in Mean, Typical Error of Measurement (absolute and relative values), Reliability Coefficients (Reliability) and Intra Correlation Coefficients (ICC). Values in brackets represent the 95% Confidence Limits.

**Fig 5:**
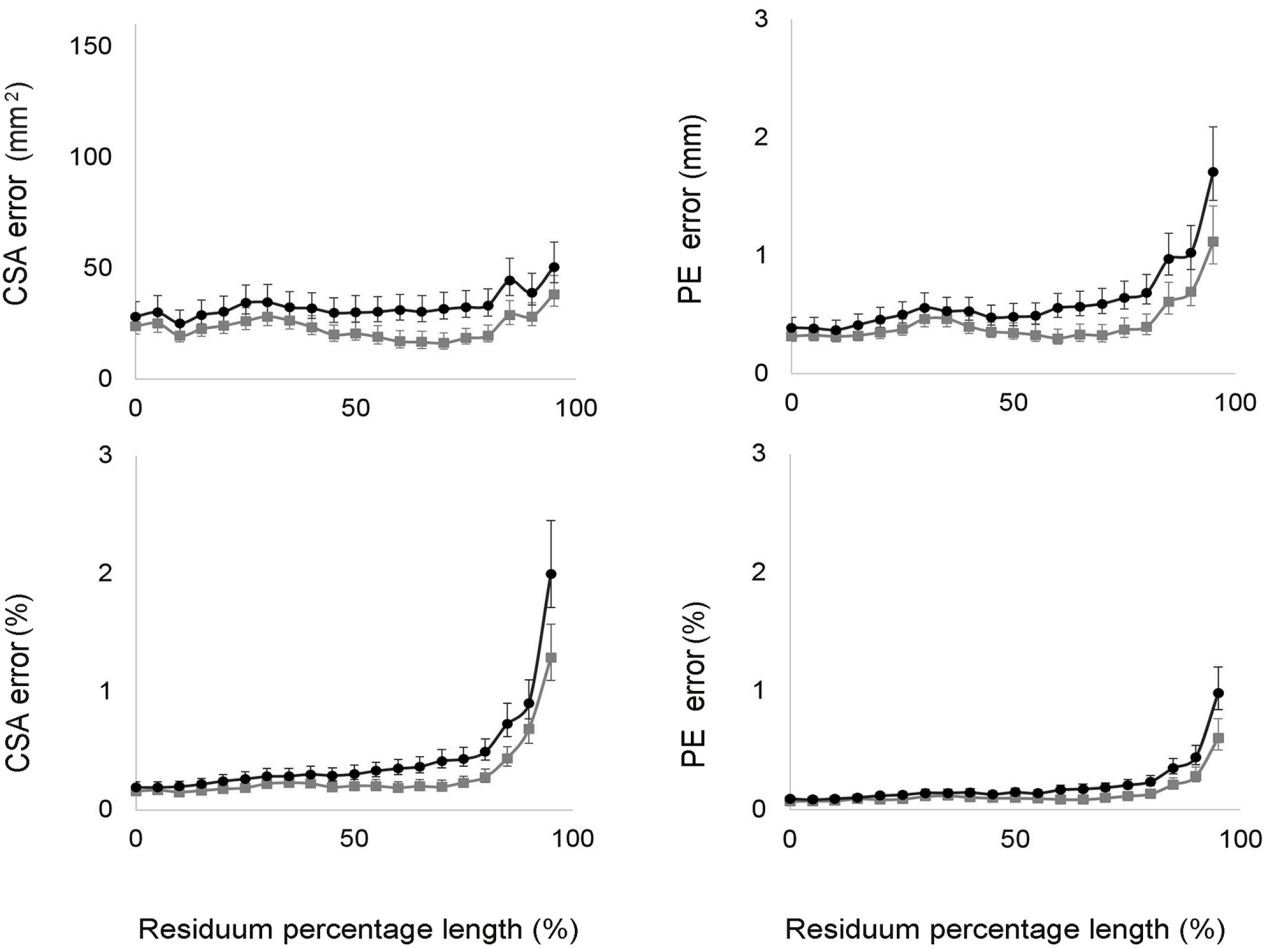
Residual limb shape reliability results. The graphs represent the TEMs and the lower and upper CL for CSAs and PE calculated for each section along the residual limb model, and expressed in absolute units and as percentage of the Romer scanner’s original measure. Grey lines indicate intra-reliability results. Black lines indicate inter-reliability results (0% indicates the first proximal section of the residual limb model).

Results regarding the residual limb shape in term of CSA and PE revealed that the inter-observer error was always higher compared with the intra-observer error. As shown in Fig 5, CSA and PE errors (TEMs) were always higher when considering inter-rater reliability, and they increased exponentially beyond 75% of the length of the residual limb.

## Discussion

The ability to accurately and reliably quantify residual limb shape and volume, together with changes in these variables over time, is important to help decision making in timing and design of prosthetic sockets. This study assessed the validity and reliability of a new structured light 3D scanner for measuring lower-limb residual limb volume and shape characteristics in a range of residual lower-limb models.

Validity and reliability determine the accuracy of a new measurement instrument and are important in terms of the quality of the data and subsequent future clinical application [35]. Because no variance in the residual limb model volume occurs, the technical error of the Artec Eva scanner could be evaluated, before commencing further research on human residual limbs.

This investigation is the first to include both transtibial and transfemoral residual limb models to assess the accuracy of a new structured light 3D scanner for residual limb volume and shape monitoring. In addition, this study is particularly timely given the increased number of emerging CAD/CAM technologies socket volume accommodation.

The Artec Eva scanner showed a high degree of relative validity (<2%) in volume measurements with bias values of <1% when considering only transfemoral residual limb models (Fig 2). These results are consistent with the literature investigating CAD systems for residual limb volume measurements [18, 19, 23, 36].

The use of high precision and resolution laser scanner has been suggested for evaluation of new scanning systems [4], and in this study the Romer laser scanner (with an accuracy of one tenth of a millimetre), was selected as the criterion measure to assess the validity of the Artec Eva 3D scanner on rigid residual limb models. Previous validity assessments have been limited by the fact that, in most cases, no gold standard more accurate than the instrument under development was available [4]. One of the most accurate CAD systems investigated, the CAPOD laser scanner reported variations in validity between 0.3 and 2.5% of water displacement (criterion measure) [18, 19]. Similar results were found for the VIUScan [23] marker-assisted laser scanner, and the Go!SCAN 3D structured white light scanner, with 0.5% and 1.1% of the criterion (a single 3D printed transtibial model of known geometry). High levels of accuracy (<2%) were also reported for the TracerCAD system in gross volume and lengths on a cylindrical model [36]. However, this system was not as consistent when applied to the more complex transtibial residual limb model [37].

A very small magnitude for RMSE (< 1mm) was measured with the Artec Eva scanner. When comparing models collected with the Artec Eva and Romer scanners, the average distance between models was always lower than 1 mm (0.6% of a diameter of 160 mm), with the highest differences highlighted at the prominences of the residual limb models, including the tibial tuberosity and cut end of the tibia for the transtibial models and lateral aspect of cut end of the femur for the transfemoral models (see Fig 3). Results were similar to those previously reported [23] for the VIUScan and Go!SCAN systems, which both had a surface length error magnitude up to 0.20 mm and 0.33 mm, respectively. Similar values were observed in the trend for increasing bias of the CSAs along the residual limb (higher discrepancies at the proximal end of the model up to 2% for the CSA and up to 1% for the PE).

Reliability results showed that the Artec Eva scanner is a very reliable instrument for lower-limb residual limb volume and shape measurements. Correlation coefficients (ICCs) both for intra- and inter-rater repeatability exceeded the 0.90 threshold for clinically relevant reliability [32, 38]. The inter-observer error (between different observers) was higher than the intra-observer error (within the same observer). In fact, reliability coefficients for the Artec Eva scanner increased when different observers performed the scans. However, these coefficients were 58% (for inter-rater coefficient) and 69% (for intra-rater coefficient) lower compared to the ones reported when scanning residual limb models with the Omega Tracer scanner (45 ml, ~5%), currently considered as the most reliable scanner for residual limb volume monitoring in applied clinical practice [2, 22]. An intra-observer reliability coefficient of ~14 ml (0.5%) and inter-observer reliability coefficient of ~19 ml (0.7%) for the Artec Eva scanner indicates that there is a 95% chance that a next measurement will fall within 14 ml of the initial measurement, independent of session/occasion and within 19 ml of the initial measurement, independent of observer. Both values are important in terms of the ability to detect meaningful changes in residual limb volume and the flexibility to switch between clinical observers.

When expressing typical errors within and between observers as percentage of volumes (0.2 and 0.3% respectively), they were lower compared to other studies investigating different 3D CAD systems (repeatability was 0.4% for the CAPOD system [18] and 3% for the TracerCAD [39]). Similar to the Artec Eva scanner, the laser VIUScan estimated repeatability coefficient was higher between observers, than within observers. However these results were restricted to transtibial models. The Artec Eva scanner was found to be reliable, not only for volume measurements, but also for residual limb shape assessment. Model CSA and PE errors were always lower than 1% for most of the length of the residual limb (both for intra and inter-assessment reliability), with future advantages for assessing complex traumatic residual limbs. In addition, model sizes and BCOM estimation showed errors always lower than 0.2%. This information can be useful for the estimation of inertial parameters and future biomechanical investigations related to patient gait characteristics.

Additional advantages of the Artec Eva scanner include the fact that it is considerably faster and less expensive than other imaging systems. Also, the Artec Eva scanner can detect colours, allowing the identification of anatomical reference points on the residual limb skin surface of the patient. This feature could be particularly useful during longitudinal assessment of residual limb change and alignment processes.

As previously stated, the Artec Eva scanner has some potentially useful features for monitoring amputee residual limb shapes and volumes. However, some limitations need to be considered. The definition of the residual limb proximal end is based on three landmarks that could generate sections, which are not transverse to the approximate axis of the residuum shape. Future tests on human amputees should be performed adopting the anatomical landmarks suggested by Geil in 2007 [17] (including the mid patellar tendon) or by Bolt in 2010 [9], who adopted the knee joint reference points to define the proximal end of the residuum. Although the scanning conditions were always the same and the observers were all trained the same to scan the models, the 3D models had to be manually aligned to be compared during the post-processing phase. This aligning could introduce some measurement artefacts and increase random errors or systematic errors in the RMSE, particularly at the distal end of the residual limb model. Therefore, the validity and the reliability coefficients obtained for the residual limb models may represent overestimations. Future studies should consider the methods suggested by Zachariah [40], which adopted automatic algorithms for aligning the residual limb 3D models. In this study, only 5 transtibial and 5 transfemoral models were measured, showing that the validity of the instrument possibly depends on the residual limb volume *per se*. Future studies should include different ranges of volumes and progress investigations to human amputees. As demonstrated also in previous studies [9, 22], it is therefore highly likely that instrument validity and reliability coefficients will increase when the Artec Eva scanner is applied to amputee residual limbs, primarily because of movement artefacts, different characteristics of the patients (size, skin features), post-doffing changes and different skin colours, contours and surfaces.

## Conclusion

The Artec Eva scanner is a valid and reliable method for assessing the shape and volume of residual lower-limb models. Indeed, the mean percentage error of the Artec Eva scanner (validity) was only 1.4%, compared to the volumes measured using the criterion method. This reflects the results of the most accurate methods presented in the literature^9^ and current scanning technologies. Furthermore, intra and inter-rater reliability coefficients were 0.5% and 0.7%, which are considerably less than the values reported current 3D scanners used in clinical practice (~5%), when scanning residual limb models. The Artec Eva scanner was also valid and reliable for assessing other residual limb model dimensions, centre of mass and cross sectional area. This measurement system still needs to be tested on human residual limbs and the results need to be compared with the current system used on amputees. However, the method has the potential to i) quantify smaller volume fluctuations (e.g. within the day fluctuations) ii) identify differences in the shrinking process between patient groups (transtibial/transfemoral—traumatic vs vascular causes) iii) provide shape information, which can help to understand where the residual limb changes are greatest and iv) provide texture/colour information, which might be useful to monitor the residual limb after surgery.

## Supporting information

S1 Table Strengthening the Reporting of Observational Studies in Epidemiology (STROBE)–STROBE checklist. (DOC)
